# Association between blood neurofilament light chain levels and vascular cognitive impairment: a systematic review and meta-analysis

**DOI:** 10.3389/fnins.2026.1779717

**Published:** 2026-02-20

**Authors:** Fu-li Qin, Xia He, Xia-lian Huang, Yan-qiu Wang, Feng-le Mao, Ke-fu Ding

**Affiliations:** 1 School of Health Preservation and Rehabilitation, Chengdu University of Traditional Chinese Medicine, Chengdu, China; 2 Sichuan Provincial Bayi Rehabilitation Center (Sichuan Provincial Rehabilitation Hospital), Chengdu, China; 3 Pacific Care Home, Chengdu, China

**Keywords:** blood NfL, cognitive function, meta-analysis, systematic review, vascular cognitive impairment

## Abstract

**Objective:**

Vascular cognitive impairment (VCI) is the second leading cause of cognitive impairment after Alzheimer’s disease, primarily associated with vascular risk factors and cerebrovascular disease. Advances in ultra-low concentration single-molecule array (Simoa) technology have enabled the quantitative monitoring of blood neurofilament light chain (NfL) levels. Consequently, we performed a meta-analysis to evaluate the association between blood NfL levels in VCI.

**Methods:**

This meta-analysis was conducted in accordance with the PRISMA guidelines. We systematically searched PubMed, Embase, Cochrane Library, Web of Science, China National Knowledge Infrastructure (CNKI), VIP Information (VIP), and Wanfang Data databases, with a search period extending from database inception to December 3, 2025. Two reviewers independently performed the literature selection, data extraction, and assessed the study quality using the Newcastle-Ottawa Scale (NOS). The weighted mean difference (WMD) and its 95% confidence interval (CI) were used to combine effect sizes. Heterogeneity was evaluated utilizing the Chi-square (*χ2*) test (Cochran’s Q) and the index of inconsistency (*I^2^*) statistic. Publication bias was evaluated by funnel plots and Egger’s regression analysis.

**Results:**

This systematic review included 13 studies, comprising 3,716 patients. The meta-analysis results indicated that blood NfL levels in VCI patients were significantly higher than those in the non-VCI group (WMD = 15.06, 95% CI = [11.41, 18.71], *p* < 0.00001). Subgroup analysis further demonstrated that the elevated trend of NfL levels in VCI patients remained consistent across different study designs, detection methods, VCI Subtypes, countries, control group types, specimen type, and statistical adjustment (*p* < 0.05).

**Conclusion:**

Our results suggest that blood NfL levels are significantly higher in VCI patients compared to non-VCI patients, indicating a strong association between blood NfL and VCI. This supports its potential as a discriminative biomarker for VCI.

**Systematic review registration:**

https://www.crd.york.ac.uk/prospero/, identifier CRD420251240858.

## 1 Introduction

Vascular cognitive impairment (VCI) encompasses a spectrum of cognitive disorders resulting from cerebrovascular pathologies and their associated risk factors (Wardlaw et al., 2013). The spectrum of VCI ranges from mild vascular cognitive impairment to vascular dementia (VaD) (Badji et al., 2023; Vemuri et al., 2022; Biesbroek et al., 2017), accounting for approximately 20%–40% of all dementia cases (Rundek et al., 2022). In the context of a globally aging population, VCI imposes a significant global disease burden (Yang et al., 2017; Ka, 2013; Semerano et al., 2024; GBD 2019 Dementia Forecasting Collaborators, 2022). Although the long-term course of VCI is typically progressive and substantially impairs patients’ function and quality of life (Rundek et al., 2022), early-stage VCI may benefit from active intervention targeting vascular risk factors to effectively slow cognitive decline (Iadecola et al., 2019; Livingston et al., 2020). However, the diagnosis of VCI currently relies mainly on post-symptom clinical evaluation, cognitive assessment, and neuroimaging (Ma et al., 2024; Chinese Stroke Association Vascular Cognitive Impairment Subcommittee, 2024), with a lack of effective tools for early detection (Chinese Stroke Association Vascular Cognitive Impairment Subcommittee, 2024). In addition, compared with Alzheimer’s disease, patients with VCI tend to have higher disability and mortality rates (Li et al., 2020; Gao et al., 2016; Gorelick et al., 2011), which is likely attributable to delayed identification and management of underlying cerebrovascular lesions and modifiable risk factors (Gorelick et al., 2011; Li et al., 2020; Gao et al., 2016). Consequently, there is a pressing need for highly sensitive and discriminatory biomarkers to monitor disease progression and provide early warnings (Badji et al., 2023).

Recent research has identified several blood-based biomarkers related to neuronal function that show promise for VCI (Wang Z. et al., 2021; Wang J. H. et al., 2021; Jiang et al., 2022). More importantly, such blood biomarkers offer significant practical advantages including accessibility, objectivity, minimal invasiveness, and low cost (Alcolea et al., 2023), making them uniquely promising for the adjunctive identification and diagnosis of early VCI (Ma et al., 2024; Ka Young et al., 2022). Advances in ultra-sensitive detection technologies, notably single-molecule array (Simoa) platforms, now allow reliable quantification of low-abundance, brain-derived proteins in blood (Laura et al., 2024). Neurofilament light chain (NfL) protein is a major component of the axonal cytoskeleton, expressed exclusively in neurons, and is highly specific for detecting neuronal injury and death (Khalil et al., 2018; Gaetani et al., 2019). Under normal conditions, neurons continuously release low levels of NfL, which remains relatively stable within axons and has a low turnover rate (Kern et al., 2019; Merluzzi et al., 2018). When axons in the central nervous system are damaged, NfL, as a specific byproduct of such damage, is released into the extracellular space, then enters the cerebrospinal fluid, and subsequently enters the peripheral blood circulation at lower concentrations (Peters et al., 2020; Teunissen and Khalil, 2012; Khalil et al., 2024). The levels of NfL in the blood increase proportionally with the extent of axonal injury (Olsson et al., 2019; Rajeev et al., 2022). VCI is associated with various cerebrovascular pathological changes, such as large ischemic strokes, lacunar infarcts, microinfarcts, demyelination, small artery sclerosis, cerebral amyloid angiopathy, and expansion of perivascular spaces (Vinciguerra et al., 2020; Skrobot et al., 2017). The resulting processes (Vinciguerra et al., 2020; Wallin et al., 2018; Chen et al., 2019), such as chronic cerebral hypoperfusion, blood–brain barrier disruption, and neuroinflammation, may ultimately lead to axonal degeneration and injury (Calabrese et al., 2016). Therefore, NfL serves as a potential biomarker for subcortical large-diameter axonal degeneration and neuronal damage (Teunissen and Khalil, 2012). Among various candidate biomarkers, blood NfL has thus emerged as a leading indicator of neuronal health, with elevated levels robustly linked to cognitive decline across multiple studies (Rundek et al., 2022; Meeter et al., 2016; Steinacker et al., 2016; Barro et al., 2020; Raghavan et al., 2025; Tao et al., 2025). Despite increasing evidence, dedicated systematic reviews and quantitative meta-analyses on the association between blood NfL levels and VCI are still lacking. Therefore, this study aims to systematically review and meta-analyze existing evidence on this relationship, to provide an evidence-based foundation for the association between blood NfL and VCI and its potential as a discriminative biomarker.

## 2 Methods

### 2.1 Research structure

This systematic review and meta-analysis was conducted in accordance with the 2020 Preferred Reporting Items for Systematic Reviews and Meta-Analyses (PRISMA) guidelines (Page et al., 2021). The study protocol was prospectively registered on the International Prospective Register of Systematic Reviews (PROSPERO) under registration number CRD420251240858.

### 2.2 Literature search

We conducted a comprehensive systematic search across seven databases: PubMed, Embase, the Cochrane Library, Web of Science, China National Knowledge Infrastructure (CNKI), VIP Information (VIP), and Wanfang Data. The search encompassed all literature from database inception until December 3, 2025. The strategy incorporated both controlled vocabulary (e.g., MeSH, Emtree) and free-text terms. Relevant search terms for vascular diseases included “vascular,” “stroke,” “cerebrovascular disorders,” “cerebral infarction,” “brain infarction,” “cerebral hemorrhage,” “cerebral small vessel diseases,” and other related terminology. For cognitive impairment, the search terms included “dementia,” “cognitive impairment,” “cognitive decline,” “cognitive dysfunction,” “vascular cognitive impairment,” and “vascular dementia.” For biomarkers, the search focused on terms like “neurofilament light chain,” “Neurofilament light chain protein,” and “neurofilament proteins,” with the search specifically restricted to studies conducted on plasma, serum, or blood samples. To minimize publication bias and identify all eligible data, we supplemented the electronic search by manually reviewing the reference lists of included studies and relevant reviews, as well as searching conference abstracts and preprint repositories. The detailed search strategy is provided in Supplementary material S1. Two investigators independently performed the initial screening of titles and abstracts against the predefined inclusion and exclusion criteria.

### 2.3 Literature screening process

Studies meeting the following criteria were included: (1) studies involving adults (≥18 years) clinically diagnosed with VCI or VaD, or individuals at risk of VCI/VaD; (2) studies assessing the blood (plasma or serum) NfL levels; (3) observational study designs, including cohort studies, case–control studies, or cross-sectional studies; (4) studies providing sufficient data to calculate or extract effect sizes, covering both the VCI/VaD group and the cognitively normal control group. Studies were excluded if they met the following criteria: (1) duplicated publications, reviews, meta-analyses, case reports, or animal studies; (2) studies for which the full text could not be accessed, or key data could not be extracted or calculated; (3) research not available in Chinese or English. Additionally, studies with confirmed or suspected overlapping participant cohorts were excluded to ensure sample independence in the meta-analysis.

### 2.4 Data extraction

Two investigators (FLQ and YQW) independently extracted data from each included study using a standardized, pre-piloted data extraction form. Any discrepancies between the extractors were resolved through discussion and, if necessary, by consulting a third senior researcher. The following information was systematically collected: first author, publication year, study duration, country, study design type, VCI subtype, VCI diagnostic criteria, sample type, NfL detection method, statistical adjustment, age, gender composition, sample size, and NfL levels. To ensure comparability across all studies and to maintain a consistent cross-sectional analytical framework, we extracted blood NfL levels from the first available measurement point (baseline or initial assessment) for all analyses, even if a study reported longitudinal follow-up data. For studies reporting NfL concentrations as median with range or interquartile range, we estimated the corresponding mean and standard deviation using validated statistical methods described by Luo et al. (2018). All included studies reported the key data necessary for meta-analysis in a complete and clear manner. Therefore, no requests for additional data were made to the authors of the included studies.

### 2.5 Quality assessment

The methodological quality of each included study was assessed independently by two investigators (FLQ and YQW) using the Newcastle-Ottawa Scale (NOS) (Stang, 2010). This scale assesses studies on three dimensions: selection, comparability, and exposure/outcome assessment, with a total of 8 items and a maximum possible score of 9. In line with common practice, studies scoring 6 or more points were of high quality. Any discrepancies in scoring were resolved through discussion, with adjudication by a third senior researcher if consensus could not be reached.

### 2.6 Statistical analysis

The statistical analysis for this study was performed using Review Manager 5.4 software. Since blood NfL levels are continuous variables, Weighted Mean Difference (WMD) along with their 95% Confidence Intervals (CIs) were used as the combined effect size. Effect sizes from individual studies were pooled using inverse-variance (IV) weighting. The heterogeneity between studies was assessed using the Chi-square test (Cochran’s Q) and the inconsistency index (*I^2^*). If the results indicated low heterogeneity (*p* > 0.05 or *I^2^* ≤ 50%), a fixed-effect model was used for the meta-analysis. Conversely, if high heterogeneity was indicated (*p* < 0.05 or *I^2^* > 50%), a random-effects model was used. The combined effect size was presented using a forest plot, and *p* < 0.05 was considered statistically significant.

### 2.7 Subgroup analysis

Given the limited number of included studies (*n* = 13), we conducted exploratory subgroup analyses to explore potential sources of heterogeneity and assess the robustness of the primary findings (Higgins et al., 2022). The pre-specified subgrouping dimensions included study design, VCI subtype, method of NfL quantification, geographical region, control group type, specimen type, and statistical adjustment. Meta-analyses within each subgroup were performed using the inverse-variance (IV) weighted random-effects model. Statistical significance of differences between subgroups was assessed using the Chi-square test, with *p* < 0.05 considered significant. All subgroup findings should therefore be interpreted with caution as hypothesis-generating.

### 2.8 Sensitivity analysis

To evaluate the influence of each included study on the combined effect in the presence of significant heterogeneity, the leave-one-out method was employed in this study.

### 2.9 Publication bias

When the number of included studies was ≥10, Egger’s regression test was performed using Stata 12.0, and a funnel plot was generated using Review Manager 5.4 to assess publication bias (Egger et al., 1997).

## 3 Results

### 3.1 Results of study inclusion

**Figure 1:**
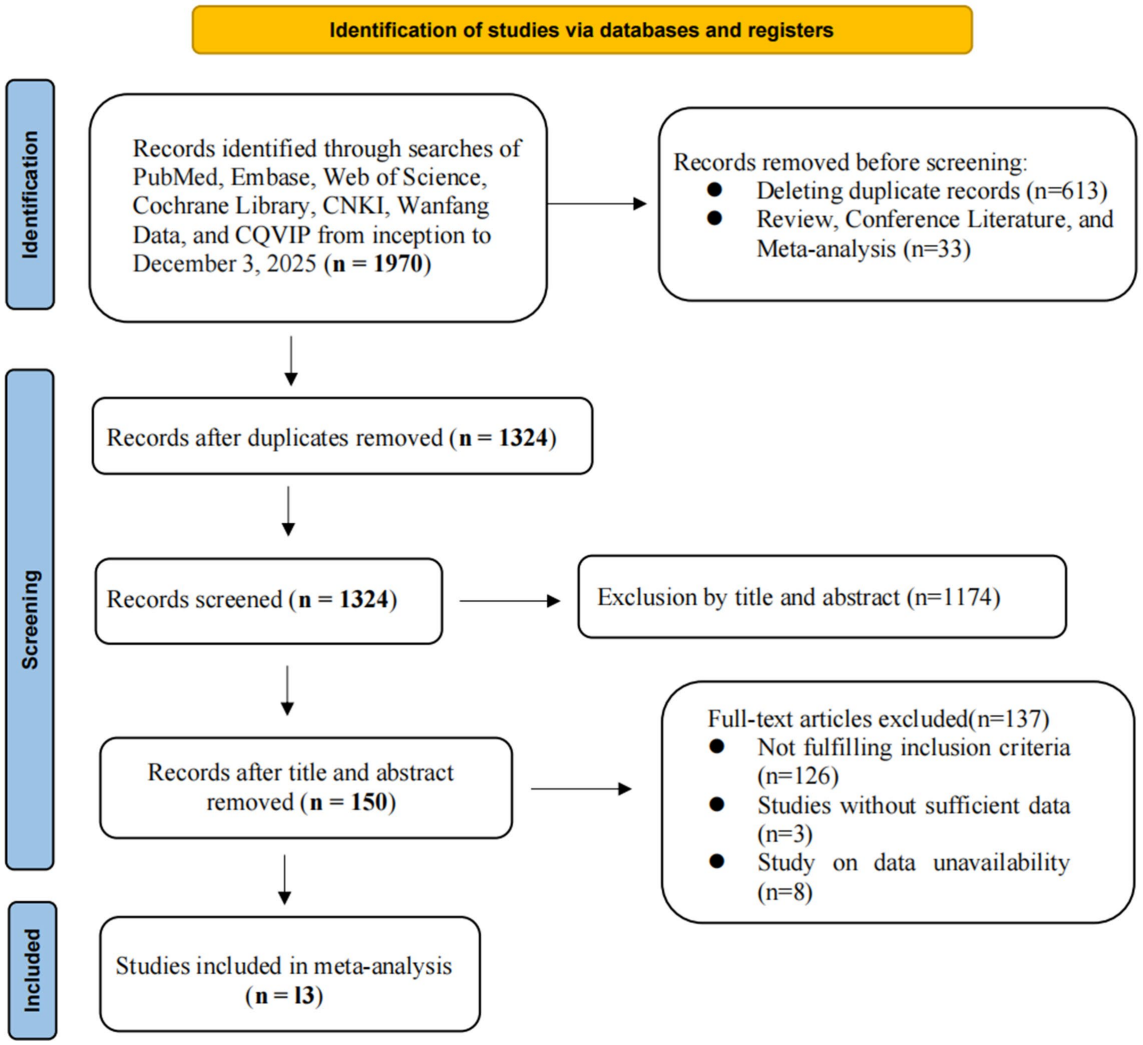
The preferred reporting items for systematic reviews and meta-analyses flow diagram for study selection.

We retrieved a total of 1970 articles through systematic searches, including 324 from PubMed, 764 from Embase, 522 from Web of Science, 266 from the Cochrane Library, 17 from CNKI, 61 from Wanfang, and 15 from VIP. After excluding 613 duplicate articles, 150 potentially relevant articles were identified through initial screening of titles and abstracts. Following full-text review and data extraction, 13 eligible studies were ultimately included (Wang Z. et al., 2021; Wang J. H. et al., 2021; Jiang et al., 2022; Trieu et al., 2024; Ming et al., 2022; Sanchez et al., 2024; Chong et al., 2023; Ma et al., 2020; Chua et al., 2023; Lee et al., 2025; Yue et al., 2024; Gaur et al., 2025; Sun et al., 2025), involving 3,716 patients in total. Figure 1 shows the flow diagram of the systematic search and selection process.

### 3.2 Basic characteristics of included studies

Table 1 presents the main characteristics of the studies included. Among them, 9 studies were prospective cohort studies (Wang Z. et al., 2021; Wang J. H. et al., 2021; Jiang et al., 2022; Trieu et al., 2024; Ming et al., 2022; Sanchez et al., 2024; Lee et al., 2025; Yue et al., 2024; Gaur et al., 2025), 3 were cross-sectional studies (Chong et al., 2023; Ma et al., 2020; Sun et al., 2025), and 1 was a case–control study (Chua et al., 2023). The included studies were primarily published between 2020 and 2025, and data were mainly collected between 2014 and 2023. The study participants were primarily elderly, averaging over 60 years of age. Of the 13 included studies, 10 adjusted for potential confounders such as age, sex, and educational level in their analyses, while 3 did not perform such adjustments. The studies mainly came from China (Wang Z. et al., 2021; Wang J. H. et al., 2021; Jiang et al., 2022; Ming et al., 2022; Ma et al., 2020; Yue et al., 2024; Sun et al., 2025) (*n* = 7), while the remaining studies from the Netherlands (Trieu et al., 2024), Canada (Sanchez et al., 2024; Gaur et al., 2025), Singapore (Chong et al., 2023; Chua et al., 2023), and South Korea (Lee et al., 2025). All studies measured NfL levels in blood, with 10 studies using the Single Molecule Array (Simoa) technology (Wang Z. et al., 2021; Wang J. H. et al., 2021; Jiang et al., 2022; Trieu et al., 2024; Sanchez et al., 2024; Chong et al., 2023; Ma et al., 2020; Chua et al., 2023; Lee et al., 2025; Sun et al., 2025), 2 using Enzyme-Linked Immunosorbent Assay (ELISA) (Ming et al., 2022; Gaur et al., 2025), and 1 using Electrochemiluminescence Immunoassay (ECLIA) (Yue et al., 2024). The detection sample types included plasma (*n* = 8) and serum (*n* = 5). The diagnosis of VCI was primarily based on multiple neuropsychological assessments and clinical diagnostic criteria, including but not limited to the Montreal Cognitive Assessment (MoCA), Clinical Dementia Rating (CDR), and vascular dementia-related diagnostic criteria (e.g., National Institute of Neurological Disorders and Stroke—Association Internationale pour la Recherche et l’Enseignement en Neurosciences, NINDS-AIREN; Gorelick criteria; Vascular Behavioral and Cognitive Disorders, VASCOG). The bias risk for the 13 included studies was assessed using the NOS, with a median score of 8 (range: 7–9, Table 2). Among the 9 prospective cohort studies, the NOS scores ranged from 7 to 9 (median 8), the 3 cross-sectional studies had scores from 8 to 9 (median 9), and the 1 case–control study scored 8. According to the widely adopted classification criteria of the scale (total score ≥7 is generally considered high quality), all included studies were regarded as high-quality studies.

**Table 1:** Characterization of the studies included in the systematic review.

Author/year	Study period	Country	Study design	VCI subtypes	Diagnosis of VCI	Specimen	NfL detection methods	Statistical adjustment
Ming et al. (2022)	2018–2020	China	Prospective cohort study	PSCI	MoCA	Serum	ELISA	Unadjusted ^a^
Yue et al. (2024)	2020–2023	China	Prospective cohort study	PSCI	MoCA	Serum	ECLIA	Unadjusted ^b^
Trieu et al. (2024)	2014–2018	Netherlands	Prospective cohort study	VCI	CDR, MMSE	Plasma	Simoa	Adjusted ^c^
Sanchez et al. (2024)	2014–2017	Canada	Prospective cohort study	PSCI	MoCA	Plasma	Simoa	Adjusted ^d^
Chong et al. (2023)	2016–2019	Singapore	Cross-sectional study	VaD	Memory clinic diagnoses	Plasma	Simoa	Adjusted ^e^
Wang J. H. et al. (2021)	2016–2019	China	Longitudinal cohort study	P-SCI	TICS-40	Serum	Simoa	Adjusted ^f^
Ma et al. (2020)	2018–2020	China	Prospective cross-sectional study	VaD	NINDS-AIREN, DSM-5	Serum	Simoa	Adjusted ^g^
Chua et al. (2023)	NA	Singapore	Case–control study	VaD	NINDS-AIREN	Plasma	Simoa	Unadjusted ^h^
Lee et al. (2025)	2018–2022	South Korea	Prospective cohort study	CADASIL-CI	K-MMSE	Serum	Simoa	Adjusted ^i^
Gaur et al. (2025)	NA	Canada	Prospective cohort study	vMCI	Gorelick	Plasma	hs-ELISA	Adjusted ^j^
Sun et al. (2025)	2017–2020	China	Cross-sectional study	SIVD	VASCOG	Plasma	Simoa	Adjusted ^k^
Wang Z. et al. (2021)	2017–2019	China	Prospective cohort study	PSCI	MoCA	Plasma	Simoa	Adjusted ^l^
Jiang et al. (2022)	2017–2019	China	Prospective cohort study	PSCI	MoCA	Plasma	Simoa	Adjusted ^m^

Author/year	Patients with VCI				Control group (cognitively normal)			
	Mean age	Male/female	Sample size	NfL (pg/mL)	Mean age	Male/female	Sample size	NfL (pg/mL)
Ming et al. (2022)	69.53 ± 5.72	52/37	89	75.97 ± 9.93	68.97 ± 5.89	71/46	117	60.59 ± 11.42
Yue et al. (2024)	66.84 ± 9.27	21/18	39	72.94 ± 25.07	63.90 ± 11.13	27/34	61	50.32 ± 14.48
Trieu et al. (2024)	68.8 ± 8.6	96/58	154	26.8 ± 21.4	65.7 ± 7.5	68/60	128	16.5 ± 7.2
Sanchez et al. (2024)	69.2 ± 7.4	110/51	161	27.7 ± 20.5	66.7 ± 6.0	33/11	44	17.5 ± 5.6
Chong et al. (2023)	75 ± 9	14./8	22	33.5 ± 22.98	74 ± 6	27/16	43	20.0 ± 9.20
Wang J. H. et al. (2021)	65.18 ± 8.61	26/23	49	75.64 ± 17.22	64.86 ± 9.37	148/107	255	49.47 ± 24.08
Ma et al. (2020)	69.27 ± 6.18	60/36	96	19.26 ± 4.71	69.59 ± 5.31	49/31	80	8.49 ± 2.37
Chua et al. (2023)	74.0 ± 8.3	33/17	50	43.8 ± 39.00	69.6 ± 7.0	45/48	93	15.7 ± 6.55
Lee et al. (2025)	66.7 ± 10.8	33/26	59	17.95 ± 9.51	65.9 ± 10.5	33/26	59	14.37 ± 8.07
Gaur et al. (2025)	64.2 ± 6.9	33/6	39	170.1 ± 56.1	64.0 ± 5.9	33/6	39	185.1 ± 56.7
Sun et al. (2025)	70.63 ± 6.19	36/18	54	21.85 ± 10.00	67.04 ± 6.51	14/13	27	11.23 ± 7.85
Wang Z. et al. (2021)	66 ± 13.73	538/491	1,029	55.96 ± 26.82	62 ± 9.66	355/310	665	35.73 ± 13.05
Jiang et al. (2022)	66.94 ± 15.04	55/46	101	56.41 ± 29.02	60.60 ± 16.46	102/61	163	31.26 ± 17.75

CADASIL, Cerebral Autosomal Dominant Arteriopathy with Subcortical Infarcts and Leukoencephalopathy; CDR, Clinical Dementia Rating; CNKI, China National Knowledge Infrastructure; DSM-5, Diagnostic and Statistical Manual of Mental Disorders, Fifth Edition; ECLIA, Electrochemiluminescence Immunoassay; ELISA, Enzyme-Linked Immunosorbent Assay; FIM cognitive, Functional Independence Measure cognitive subscore; hs-ELISA, High-Sensitivity Enzyme-Linked Immunosorbent Assay; ICD-11, International Classification of Diseases, Eleventh Revision; K-MMSE, Korean version of Mini-Mental State Examination; MMSE, Mini-Mental State Examination; MoCA, Montreal Cognitive Assessment; NA, Not Available; NfL, Neurofilament Light Chain; NINDS-AIREN, National Institute of Neurological Disorders and Stroke–Association Internationale pour la Recherche et l’Enseignement en Neurosciences; NOS, Newcastle-Ottawa Scale; PSCI, Post-Stroke Cognitive Impairment; PRISMA, Preferred Reporting Items for Systematic Reviews and Meta-Analyses; PROSPERO, International Prospective Register of Systematic Reviews; P-SCI, Post-Stroke Subjective Cognitive Impairment; Simoa, Single Molecule Array; SIVD, Subcortical Ischemic Vascular Dementia; SMD, Standardized Mean Difference; TICS-40, Telephone Interview for Cognitive Status-40; VaD, Vascular Dementia; VASCOG, Vascular Behavioral and Cognitive Disorders criteria; VCI, Vascular Cognitive Impairment; VIP, VIP Information; vMCI, Vascular Mild Cognitive Impairment; WMD, Weighted Mean Difference.

a Unadjusted (age not included in the primary logistic regression model).

b Unadjusted (age and sex not included in the primary linear regression model).

c Adjusted for age, sex, education, and study site in ANCOVA and linear mixed models.

d Adjusted via analysis of age/sex/education-residualized cognitive scores.

e Adjusted for age and sex in primary regression models.

f Adjusted for age and sex in the final multiple logistic regression model.

g Adjusted for age, sex, education, and multiple vascular risk factors in the final multiple regression model.

h Unadjusted (age not included in the primary logistic regression model).

i Adjusted for age, body mass index, and estimated glomerular filtration rate in cross-sectional and longitudinal models.

j Adjusted a priori for age, sex, and education in multivariable models.

k Adjusted for age and sex in analysis of covariance (ANCOVA).

l Adjusted for age, sex, education, and clinical stroke factors in multivariate logistic regression.

m Adjusted for age, sex, education, and key stroke-related factors in multivariate logistic regression.

**Table 2:** Risk of bias assessment according to the Newcastle-Ottawa Scale.

Reference	Study design	Selection	Comparability	Exposure/outcome	Total
Ming et al. (2022)	Prospective cohort	★★★★	★	★★	7
Yue et al. (2024)	Prospective cohort	★★★★	★	★★	7
Trieu et al. (2024)	Prospective cohort	★★★★	★★	★★	8
Sanchez et al. (2024)	Prospective cohort	★★★★	★★	★★★	9
Chong et al. (2023)	Cross-sectional	★★★★	★★	★★★	9
Wang J. H. et al. (2021)	Longitudinal cohort	★★★★	★★	★★★	9
Ma et al. (2020)	Prospective cross-sectional	★★★★	★★	★★	8
Chua et al. (2023)	Case–control study	★★★★	★★	★★	8
Lee et al. (2025)	Prospective cohort	★★★	★	★★★	8
Gaur et al. (2025)	Prospective cohort	★★★	★	★★★	7
Sun et al. (2025)	Cross-sectional	★★★	★	★★★	8
Wang Z. et al. (2021)	Prospective cohort	★★★★	★★	★★★	9
Jiang et al. (2022)	Prospective cohort	★★★★	★★	★★★	9

★★★★: 4 points; ★★★: 3 points; ★★: 2 points; ★: 1 point.

### 3.3 The results of meta-analysis

**Figure 2:**
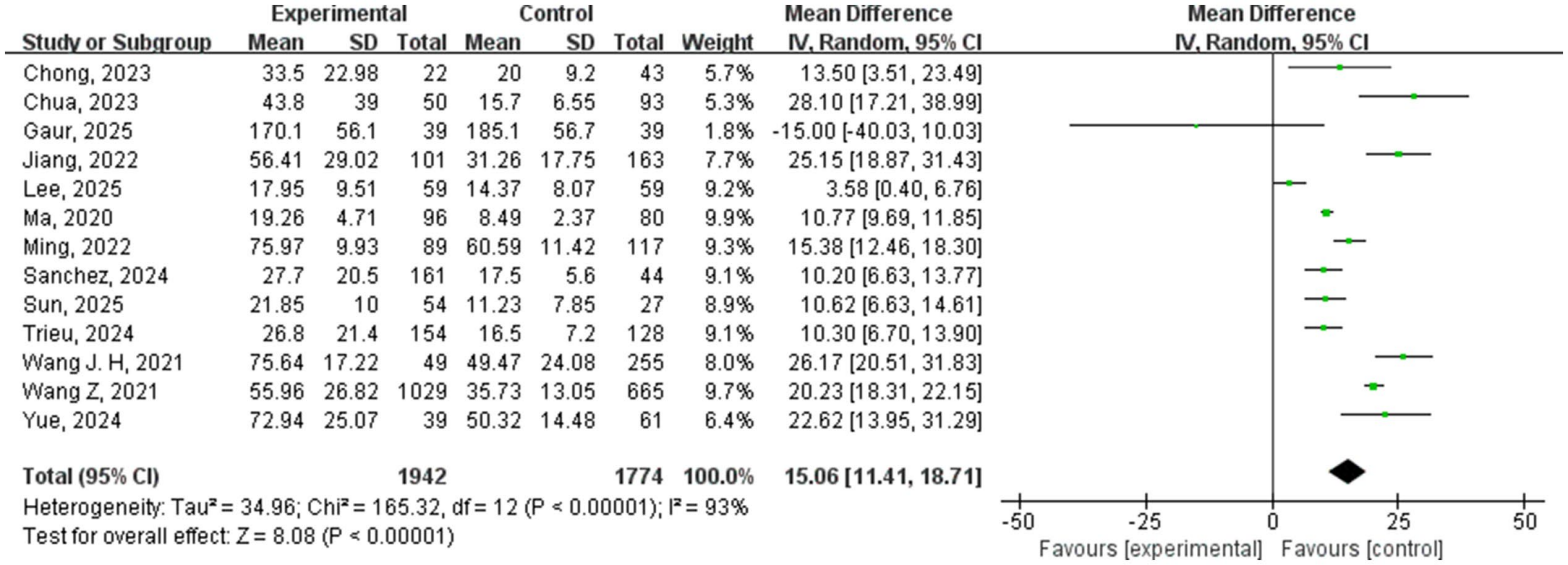
Forest plot of blood NfL levels. Each green square represents a study’s WMD, horizontal line shows 95% CI; size indicates study weight. The bottom black diamond is the pooled WMD (95% CI) from the random-effects meta-analysis of 13 studies. The dashed line at zero indicates no difference. CI, confidence interval; *I^2^*, I-squared statistic (heterogeneity); IV, inverse variance; SD, standard deviation; WMD, weighted mean difference.

A total of 13 studies compared the blood NfL levels between VCI patients (*n* = 1942) and non-VCI patients (*n* = 1774). The meta-analysis results showed that the blood NfL levels in the VCI group were significantly higher than in the non-VCI group (WMD = 15.06, 95% CI = [11.41, 18.71], *p* < 0.00001) (Figure 2), with significant statistical heterogeneity (*I^2^* = 93%, *p* < 0.00001).

### 3.4 Subgroup analysis

The meta-analysis showed high heterogeneity (*I^2^* = 93%). To explore the source of heterogeneity, we conducted subgroup analyses (Table 3). First, based on the study design, the subgroup analysis showed that blood NfL levels were significantly higher in the VCI group than in the non-VCI group in prospective cohort studies (WMD = 15.27, 95% CI = [9.98, 20.55], *p* < 0.00001, *I^2^* = 94%), cross-sectional studies (WMD = 10.79, 95% CI = [9.76, 11.82], *p* < 0.00001, *I^2^* = 0%), and case–control studies(WMD = 28.10, 95% CI = [17.21, 38.99], *p* < 0.00001). However, there were significant between-group differences between the study designs (*p* = 0.002). Secondly, subgroup analysis based on detection methods and Specimen types showed no statistically significant difference in the effect sizes between studies using Simoa technology (WMD = 15.13, 95% CI = [10.94, 19.31], *p* < 0.00001, *I^2^* = 94%) and those using other detection methods (WMD = 13.98, 95% CI = [3.15, 24.82], *p* = 0.01, *I^2^* = 76%) (*p* value for subgroup difference = 0.85). Similarly, there was no significant difference in the pooled effect sizes between serum samples (WMD = 14.89, 95% CI = [9.26, 20.52], *p* < 0.00001, *I^2^* = 94%) and plasma samples (WMD = 15.17, 95% CI = [10.03, 20.31], *p* < 0.00001, *I^2^* = 89%) (*p* value for subgroup difference = 0.94). Additionally, subgroup analyses based on VCI subtypes (post-stroke VCI subgroup (WMD = 19.40, 95% CI = [14.87, 23.93], *p* < 0.00001, *I^2^* = 88%) versus non-stroke VCI subgroup (WMD = 10.28, 95% CI = [6.53, 14.04], *p* < 0.00001, *I^2^* = 81%)), country (China subgroup (WMD = 18.18, 95% CI = [13.43, 22.93], *p* < 0.00001, *I^2^* = 95%) compared to other countries subgroup (WMD = 10.38, 95% CI = [4.91, 15.86], *p = 0*.0002, *I^2^* = 82%)), control group type (vascular risk/disease controls (cognitively normal) (WMD = 21.07, 95% CI = [16.85, 25.28], *p* < 0.00001, *I^2^* = 78%) as opposed to cognitively normal healthy controls (WMD = 9.36, 95% CI = [6.82, 11.90], *p* < 0.00001, *I^2^* = 72%)), and statistical adjustment (adjusted subgroup (WMD = 12.49, 95% CI = [11.67, 13.30], *p* < 0.00001, *I^2^* = 94%) compared to unadjusted subgroup (WMD = 16.85, 95% CI = [14.16, 19.53], *p* < 0.00001, *I^2^* = 70%)) all showed that blood NfL levels in the VCI group were significantly higher than in the non-VCI group, with significant between-group differences (*p* < 0.05).

**Table 3:** Subgroup analysis of the association of blood NfL levels with VCI.

Subgroup	NfL				Between-subgroup comparison *P* value
	Study	WMD [95%CI]	*P* value	*I^2^*	
Total	13	15.06 [11.41, 18.71]	< 0.00001	93%	
Study design					0.002
Prospective cohort study	9	15.27 [9.98, 20.55]	< 0.00001	94%	
Cross-sectional study	3	10.79 [9.76, 11.82]	< 0.00001	0%	
Case–control study	1	28.10 [17.21, 38.99]	< 0.00001	NA	
Detection methods					0.85
Simoa	10	15.13 [10.94, 19.31]	< 0.00001	94%	
Others	3	13.98 [3.15, 24.82]	0.01	76%	
VCI Subtypes					0.002
Post-stroke VCI	6	19.40 [14.87, 23.93]	< 0.00001	88%	
Non-stroke VCI	7	10.28 [6.53, 14.04]	< 0.00001	81%	
Country					0.04
China	7	18.18 [13.43, 22.93]	< 0.00001	95%	
Other countries	6	10.38 [4.91, 15.86]	0.0002	82%	
Control group type					< 0.00001
Vascular risk/disease controls (cognitively normal)	7	21.07 [16.85, 25.28]	< 0.00001	78%	
Healthy controls (cognitively normal)	6	9.36 [6.82, 11.90]	< 0.00001	72%	
Specimen					0.94
Serum	5	14.89 [9.26, 20.52]	< 0.00001	94%	
Plasma	8	15.17 [10.03, 20.31]	< 0.00001	89%	
Statistical adjustment					0.002
Adjusted	10	12.49 [11.67, 13.30]	< 0.00001	94%	
Unadjusted	3	16.85 [14.16, 19.53]	< 0.00001	70%	

For each subgroup category, the table shows the number of studies, pooled WMD with 95% CI, within-subgroup *P* value, *I^2^*, and the *P* value for the between-subgroup comparison (test for subgroup differences). CI, confidence interval; *I*^2^, I-squared statistic (heterogeneity); NfL, Neurofilament Light chain; VCI, Vascular Cognitive Impairment; WMD, weighted mean difference.

### 3.5 Sensitivity analysis

**Figure 3:**
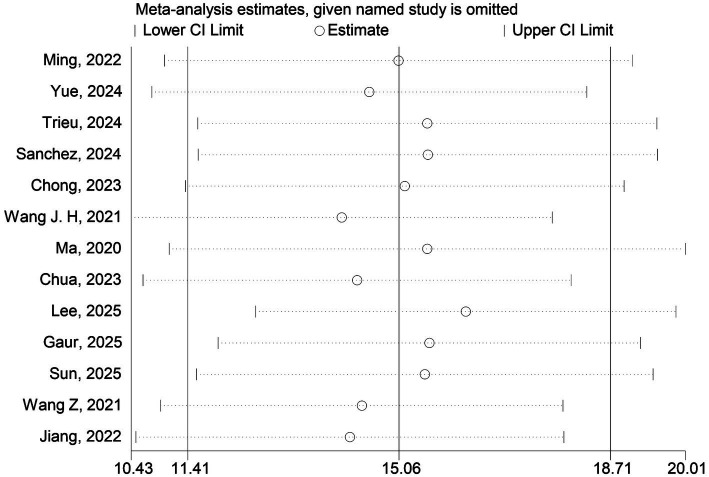
Sensitivity analysis for blood NfL levels in VCI. The plot shows the recalculated pooled WMD with 95% CI after sequentially omitting each individual study. Each point with error bars represents the pooled estimate when the corresponding study is excluded. The solid vertical line indicates the original pooled estimate (WMD = 15.06). The dashed vertical line at WMD = 0 indicates the line of no effect. CI, confidence interval.

To evaluate the stability of the meta-analysis results, we conducted a leave-one-out sensitivity analysis on the blood NfL levels to assess the influence of each study on the pooled WMD. Sensitivity analysis (Figure 3) showed that after removing any individual study, including Ming et al. (2022), Yue et al. (2024), Trieu et al. (2024), Sanchez et al. (2024), Chong et al. (2023), Wang Z. et al. (2021), Ma et al. (2020), Chua et al. (2023), Lee et al. (2025), Gaur et al. (2025), Sun et al. (2025), Wang J. H. et al. (2021), and Jiang et al. (2022). The blood NfL levels in the VCI group were significantly higher compared to those in the non-VCI group, and the pooled effect size and its 95% confidence interval did not show any directional or significant magnitude changes.

### 3.6 Publication bias

**Figure 4:**
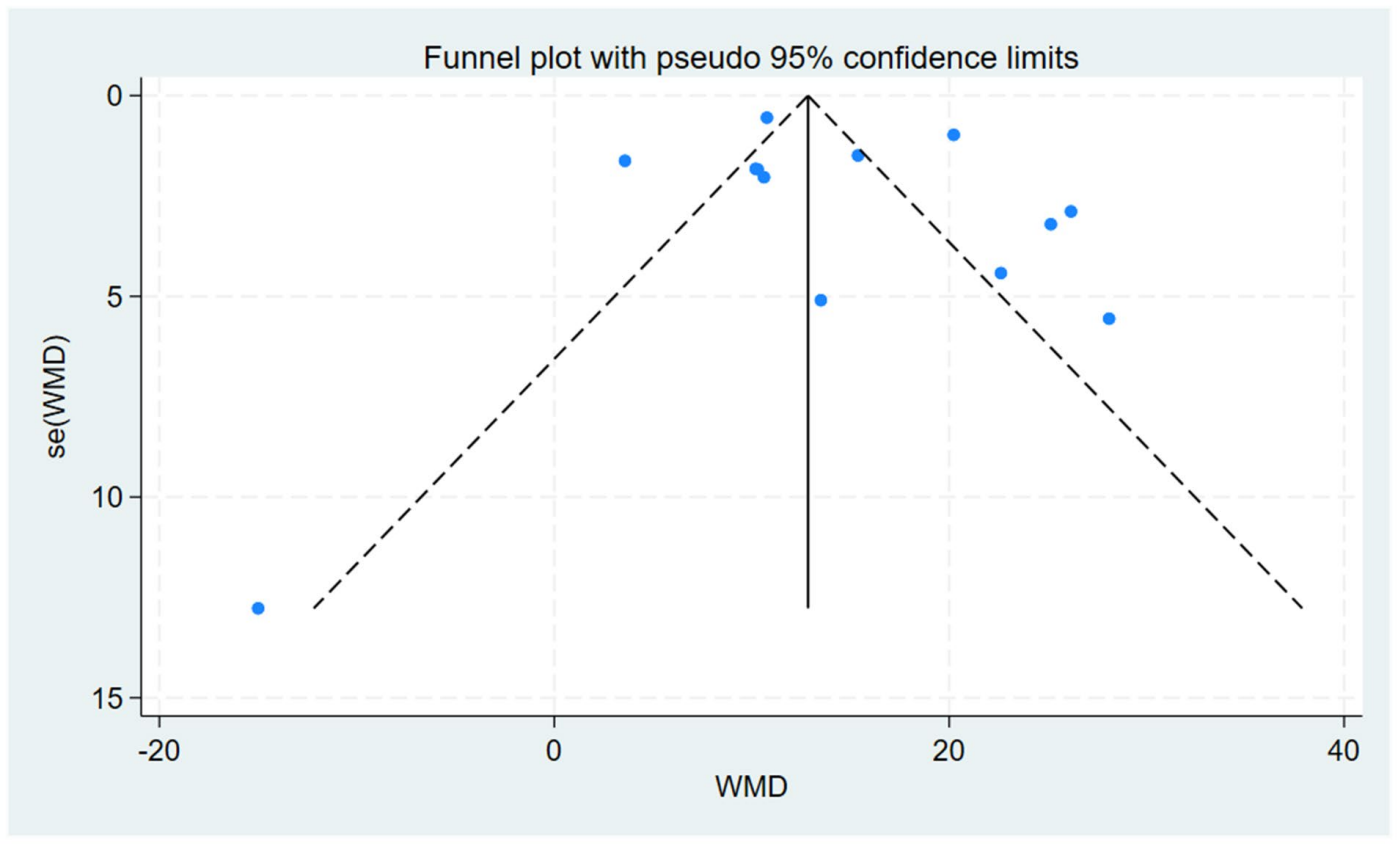
Funnel plot for NfL. Each dot represents an individual study, plotting its effect size (weighted mean difference, WMD) against its standard error (SE). The vertical line indicates the pooled effect estimate. Symmetry around this line suggests a low risk of bias.

We assessed whether there was publication bias in the results of all included studies. The results of Egger’s test (*p* = 0.8516), Begg’s test (*p* = 0.7603), and the funnel plot all indicated no potential publication bias (Figure 4).

## 4 Discussion

The main purpose of this meta-analysis is to assess the association between blood NfL levels and VCI, and to evaluate its potential as a biomarker for discriminating between individuals with and without VCI. Thirteen studies, involving 3,716 patients, were included in this meta-analysis. The meta-analysis results revealed that blood NfL levels were significantly higher in VCI patients compared to the non-VCI control group. This finding consolidates existing evidence and underscores the potential of blood NfL as a biomarker for VCI. Subgroup analyses further confirmed the robustness of this association across various study designs, VCI subtypes, detection methods, geographical regions, control group types, specimen types, and statistical adjustment.

It is currently believed that various cerebrovascular lesions and vascular risk factors are important pathological foundations for the development of VCI (Vinciguerra et al., 2020; Skrobot et al., 2017), leading to chronic cerebral hypoperfusion (Skrobot et al., 2017; Tao et al., 2025; Skrobot et al., 2016; Raghavan et al., 2025), blood–brain barrier disruption (Wallin et al., 2018; Chen et al., 2019), and neuroinflammatory responses, as well as involving processes like oxidative stress and neurotransmitter imbalance (Vinciguerra et al., 2020; Parfenov et al., 2019; Disanto et al., 2017). These pathological changes ultimately lead to axonal injury, which is the key step in the development of VCI (Vinciguerra et al., 2020; Calabrese et al., 2016). NfL has been confirmed as a reliable biomarker for axonal injury (Peters et al., 2020; Teunissen and Khalil, 2012; Rajeev et al., 2022; Duering et al., 2018; Egle et al., 2021; Olsson et al., 2016). NfL is a cytoskeletal protein (Skrobot et al., 2016; Duering et al., 2018; Egle et al., 2021) expressed exclusively in neurons and is highly enriched in the axons of neurons (Axelsson et al., 2018). NfL is not only highly specific for detecting neuronal damage and death (Peters et al., 2020; Khalil et al., 2018; Gaur et al., 2025) but also a reliable indicator of neuroinflammation and neuronal tissue damage in neurodegenerative diseases (van Ballegoij et al., 2020). Under normal conditions, NfL remains relatively stable within axons (Kern et al., 2019; Merluzzi et al., 2018). When axons are damaged, NfL is released into the extracellular space, then enters the cerebrospinal fluid, and subsequently enters the peripheral blood circulation at lower concentrations (Peters et al., 2020; Teunissen and Khalil, 2012; Khalil et al., 2024). Its levels in peripheral blood are highly correlated with cerebrospinal fluid levels (Khalil et al., 2018; Khalil et al., 2024; Disanto et al., 2017; Olsson et al., 2016) and can increase proportionally with the extent of axonal damage (Olsson et al., 2019; Rajeev et al., 2022). Therefore, the elevation of blood NfL levels is closely related to the pathological process of axonal injury (Peters et al., 2020; Teunissen and Khalil, 2012; Rajeev et al., 2022; Duering et al., 2018; Egle et al., 2021; Olsson et al., 2016).

As a sensitive biomarker for axonal injury (Duering et al., 2018), blood NfL levels are elevated in various central nervous system diseases that cause axonal damage (Chong et al., 2023; Sun et al., 2025; Duering et al., 2018; Chua et al., 2023; Wang et al., 2022; Ruoshui, 2025; Benkert et al., 2022), including vascular damage and neurodegenerative diseases like Alzheimer’s disease (AD) (Khalil et al., 2018; Khalil et al., 2024; Disanto et al., 2017; Olsson et al., 2016; Gendron et al., 2020). Studies by Sanchez et al. (2024) and Chong et al. (2023) have both found that blood NfL levels were significantly higher in VCI patients compared to non-VCI patients. This conclusion was further validated by the meta-analysis conducted by Huang et al. (2025). Additionally, research indicates that the increase in blood NfL levels is more prominent in VCI and VaD patients than in AD patients (Chong et al., 2023; Chua et al., 2023; Sun et al., 2025; Chua et al., 2023; Wang et al., 2022). This suggests that in VCI, the increase in blood NfL levels may more directly reflect axonal injury caused by the vascular lesions themselves (Rundek et al., 2022; Gaetani et al., 2019; Kern et al., 2019; Merluzzi et al., 2018; Byrne et al., 2017; Soylu-Kucharz et al., 2017). Furthermore, in the VCI animal model study by Hoyer-Kimura et al. (2021), targeted therapy was found to significantly decrease plasma NfL levels while reversing cognitive impairment, with a significant negative correlation between NfL levels and cognitive function. In conclusion, these findings are consistent with our results; NfL, as an easily detectable and sensitive blood biomarker (Alcolea et al., 2023), is significantly associated with VCI. Notably, among all the studies included, the findings of Gaur et al. (2025) are particularly noteworthy. This study focused on vascular mild cognitive impairment (vMCI), an early stage of VCI, but did not observe a significant difference in blood NfL levels between the vMCI group and the high-risk control group (individuals with significant vascular risk but normal cognition). This may be since long-term high vascular risk status or vascular disease itself can induce subclinical cerebrovascular damage and axonal injury, leading to elevated baseline blood NfL levels in individuals in the high-risk control group. Therefore, as individuals progress from the “subclinical axonal injury but cognitively normal” state to “early vMCI,” the incremental change in blood NfL levels may be relatively limited, thereby reducing its ability to early differentiate cognitive impairment in such high-risk populations. This finding reflects the potential limitations of applying blood NfL in specific high-risk populations.

Our study has several limitations. Firstly, the studies included mainly come from Asian populations, and only Chinese and English-language publications were included, which limits the generalizability of the findings to other regions, ethnicities, and linguistic backgrounds. Secondly, among the 13 studies included, 3 unadjusted for strong confounders such as age, while the remaining 10 did (Table 1). This may introduce bias into the pooled effect size and partially explain the observed significant statistical heterogeneity. Thirdly, although we attempted to explore the sources of heterogeneity through sensitivity and subgroup analyses, a considerable degree of heterogeneity remains unexplained. This suggests the potential presence of uncontrolled confounding factors. At the same time, the exploratory subgroup analyses themselves are limited by the small number of studies in each category, which reduces the statistical power to detect true differences between subgroups. Thus, while these analyses provide preliminary insights, the findings require cautious interpretation and further validation. Fourth, although most of the included studies had a prospective design (*n* = 9), the data primarily reflects the cross-sectional association between blood NfL levels and existing VCI diagnoses, rather than its predictive value. Longitudinal studies in the future are necessary to evaluate the predictive ability of baseline blood NfL levels for the occurrence or progression of VCI. Finally, while statistical tests did not indicate significant publication bias, the possibility of undetected bias inherent to meta-analyses should be considered when interpreting the pooled effect estimates. Despite these limitations, compared to the systematic review by Huang et al. (2025), this study not only significantly increased the number of included studies from 5 to 13, updating the evidence from the last 2 years, but also specifically focused on blood NfL and explored important influencing factors such as detection methods, specimen types, and statistical adjustments. Consequently, this study provides an updated systematic meta-analysis, offering important evidence-based support for the potential use of blood NfL levels as a biomarker for the differential and adjunctive diagnosis of VCI in clinical practice.

## 5 Conclusion

In conclusion, this meta-analysis provides robust evidence that blood NfL levels in VCI patients are significantly higher than those in non-VCI patients. This finding supports its potential as a discriminative biomarker and warrants future prospective studies to explore its utility in early screening and dynamic monitoring. Future large-scale, prospective, multi-center studies are needed to further validate the auxiliary diagnostic utility of blood NfL levels in VCI.

## Data Availability

The data analyzed in this study is subject to the following licenses/restrictions: This systematic review and meta-analysis are based exclusively on aggregated data extracted from previously published studies. Consequently, the availability of the raw data underlying our analyses is governed by the data-sharing policies and copyright restrictions of the respective original publications. Patient-level datasets are not publicly available through this manuscript; requests for access must be directed to the corresponding authors of the included studies, in accordance with their institutional ethical approvals and data governance frameworks. Furthermore, the included studies predominantly involved populations of Asian (primarily Chinese) ethnicity, which may limit the applicability and generalizability of our findings to other demographic and ethnic groups. Requests to access these datasets should be directed to Xiahe, xiahe163@163.com.

## Glossary

AD: Alzheimer’s disease
Aβ: Amyloid-beta
CADASIL: Cerebral autosomal dominant arteriopathy with subcortical infarcts and leukoencephalopathy
CDR: Clinical dementia rating
CI: Confidence interval
CNKI: China national knowledge infrastructure
CSF: Cerebrospinal fluid
DSM-5: Diagnostic and statistical manual of mental disorders, fifth edition
ECLIA: Electrochemiluminescence immunoassay
ELISA: Enzyme-linked immunosorbent assay
FIM-cognitive: Functional independence measure-cognitive subscale
GFAP: Glial fibrillary acidic protein
Gorelick: Gorelick vascular cognitive impairment criteria
hs-ELISA: High-sensitivity enzyme-linked immunosorbent assay
K-MMSE: Korean version of the mini-mental state examination
MMSE: Mini-mental state examination
MoCA: Montreal cognitive assessment
NA: Not applicable
NfL: Neurofilament light chain
NINDS-AIREN: National institute of neurological disorders and stroke—association internationale pour la recherche et l’enseignement en neurosciences
NOS: Newcastle-Ottawa scale
Non-CI: Control group without cognitive impairment
PD: Parkinson’s disease
PSCI: Post-stroke cognitive impairment
P-SCI: Post-stroke subjective cognitive impairment
PRISMA: Preferred reporting items for systematic reviews and meta-analyses
PROSPERO: International prospective register of systematic reviews
SIVD: Subcortical ischemic vascular dementia
Simoa: Single molecule array
SMD: Standardized mean difference
Tau: Tau protein
TICS-40: Telephone interview for cognitive status-40
VaD: Vascular dementia
VASCOG: Vascular behavioral and cognitive disorders criteria
VCI: Vascular cognitive impairment
VIP: VIP information database
vMCI: Vascular mild cognitive impairment
WMD: Weighted mean difference
