# Supplementary material for: Association between blood neurofilament light chain levels and vascular cognitive impairment: a systematic review and meta-analysis
Source: Front Neurosci. 2026 Feb 20;20:1779717. doi: 10.3389/fnins.2026.1779717 (PMC12963306; doi:10.3389/fnins.2026.1779717)
Supplement: Supplementary file 1 [file Supplementary_file_1.docx]

**Supplementary Material 1: Search Strategy for PubMed**

**Database:** PubMed
**Date Searched:** December 3, 2025
**Records Identified:**324

**Full Search Syntax:**

 (((((((((((("Blood Vessels"[Mesh]) OR "Stroke"[Mesh]) OR "Cerebrovascular Disorders"[Mesh]) OR "Cardiovascular Diseases"[Mesh]) OR "Cerebral Infarction"[Mesh]) OR "Brain Infarction"[Mesh]) OR "Cerebral Hemorrhage"[Mesh]) OR "Hemorrhage"[Mesh]) OR "Cerebral Small Vessel Diseases"[Mesh]) OR (((((((((((vascular[Title/Abstract]) OR (vessel[Title/Abstract])) OR (stroke[Title/Abstract])) OR (cerebrovascular accident[Title/Abstract])) OR (brain vascular accident[Title/Abstract])) OR (apoplexy[Title/Abstract])) OR (cerebral infarction[Title/Abstract])) OR (brain infarction[Title/Abstract])) OR (brain hemorrhage[Title/Abstract])) OR (hemorrhage[Title/Abstract])) OR (cerebral small vessel disease[Title/Abstract]))) AND ((("Dementia"[Mesh]) OR "Cognitive Dysfunction"[Mesh]) OR (((((((dementia[Title/Abstract]) OR (cognitive[Title/Abstract])) OR (cognitive impairment[Title/Abstract])) OR (cognitive decline[Title/Abstract])) OR (cognitive disorder[Title/Abstract])) OR (cognitive dysfunction[Title/Abstract])) OR (cognitive deficit[Title/Abstract])))) OR ((Vascular Cognitive Impairment[Title/Abstract]) OR (Vascular Dementia[Title/Abstract]))) AND (((((((Neurofilament Light Chain[Title/Abstract]) OR (Neurofilament Proteins[Title/Abstract])) OR (neurofilament light[Title/Abstract])) OR (NfL[Title/Abstract])) OR (NF-L[Title/Abstract])) OR (((neurofilament*[Title/Abstract]) OR (NFL[Title/Abstract])) AND (((plasma[Title/Abstract]) OR (serum[Title/Abstract])) OR (blood[Title/Abstract])))) OR (("neurofilament protein L" [Supplementary Concept]) OR "Neurofilament Proteins"[Mesh]))
